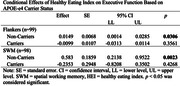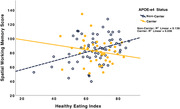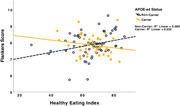# Diet, APOE‐e4, and cognition in middle‐aged women at increased risk of Alzheimer’s disease

**DOI:** 10.1002/alz.094755

**Published:** 2025-01-09

**Authors:** Chadsley M Wessinger, Kyoung Shin Park, Samantha L DuBois, Samuel Kibildis, Hadassah Som‐Pimpong, Jarod C Vance, Brittany D Armstrong, Laurie Wideman, Jennifer L Etnier

**Affiliations:** ^1^ University of North Carolina at Greensboro, Greensboro, NC USA; ^2^ Henry Ford Hospital, Detroit, MI USA

## Abstract

**Background:**

Improving modifiable risk factors, like diet quality, can protect against Alzheimer’s disease (AD). The association between diet and cognition in midlife as a function of APOE‐e4 genotype, a strong risk factor for AD, remains unknown. Therefore, this study examined the relationship between dietary intake and executive function (EF) in middle‐aged sedentary women with a family history of AD (FH+), and if the association was moderated by APOE‐e4 carrier status.

**Method:**

Baseline data from an ongoing clinical trial (NIH‐R01AG058919) were analyzed. Participants (n = 102, age = 56.9±5.8 yrs) were cognitively normal, middle‐aged women with (FH+). APOE genotype was determined from passive drool saliva samples (carriers, n = 41, non‐carriers, n = 61). Dietary data were collected using the Diet History Questionnaire III (DHQ‐III) and Healthy Eating Index (HEI), a measure of diet quality, was computed based on adherence to the Dietary Guidelines for Americans. Core EF was assessed via NIH Toolbox Flankers Test and Spatial Working Memory test (SWM). Linear regression moderation analyses were conducted using PROCESS v4.2 in SPSS including Flankers and SWM as the outcome, HEI as the predictor, APOE‐e4 carrier status as the moderator, and age, BMI, and education as covariates.

**Result:**

HEI did not predict Flankers performance after controlling for covariates (p = 0.14). However, the interaction between HEI and APOE‐e4 status trended towards significance (p = 0.05). After controlling for covariates, better HEI scores predicted better SWM performance (p<0.001). The interaction between HEI and APOE‐e4 carrier status was also statistically significant (p<0.05), suggesting APOE‐e4 carrier status moderated the relationship between HEI and SWM performance. For both the Flankers and SWM, better HEI predicted better EF in non‐carriers (p<0.05 and p<0.01, respectively) but not in carriers.

**Conclusion:**

While better HEI predicted better EF in non‐ APOE‐e4 carriers, carriers did not experience this effect, suggesting that improving diet quality may not offset the negative effects of APOE‐e4 on cognition in middle‐aged women at increased risk of AD. Future research should explore if the effects of prescriptive dietary interventions on cognition vary by APOE genotype. The content of this manuscript is solely the responsibility of the authors and does not necessarily represent the official views of the NIH.